# A Tumor Surveillance Model: A Non-Coding RNA Senses Neoplastic Cells and Its Protein Partner Signals Cell Death

**DOI:** 10.3390/ijms131013134

**Published:** 2012-10-12

**Authors:** Sung Ho Jeon, Betty H. Johnson, Yong Sun Lee

**Affiliations:** 1 Department of Life Science, Hallym University, Chuncheon 200-702, Korea; E-Mail: sjeon@hallym.ac.kr; 2 Department of Biochemistry and Molecular Biology, University of Texas Medical Branch, Galveston, TX77555, USA; E-Mail: bhjohnso@utmb.edu

**Keywords:** nc886, non-coding RNA, PKR (Protein Kinase R), tumor, surveillance

## Abstract

nc886 (= pre-miR-886 or vtRNA2-1) is a non-coding RNA that has been recently identified as a natural repressor for the activity of PKR (Protein Kinase R). The suppression of nc886 activates PKR and thereby provokes a cell death pathway. When combined with the fact that nc886 is suppressed in a wide range of cancer cells, the nc886-PKR relationship suggests a tumor surveillance model. When neoplastic cells develop and nc886 decreases therein, PKR is released from nc886 and becomes the active phosphorylated form, which initiates an apoptotic cascade to eliminate those cells. The nc886-PKR pathway is distinct from conventional mechanisms, such as the immune surveillance hypothesis or intrinsic mechanisms that check/proofread the genomic integrity, and thus represents a novel example of tumor surveillance.

## 1. Human Body Complexity and Cancer Incident Rates

Cancer is one of the major health threats worldwide. In 2011, more than 1.5 million new cancer cases occurred in the United States alone [1]. It is accepted that the molecular basis of cancer resides in mutations or epigenetic alterations that lead to the abnormal expression of oncogenes and tumor suppressors. In the multi-step tumorigenesis model [2], several mutations are accumulated before developing into invasive and metastatic malignancies.

The adult human body is comprised of approximately 10^14^ cells. To reach that number, at least 10^14^ cell division events should occur when counted from a single-celled embryo. That number would be significantly higher if we considered the massive non-pathological apoptotic events that normally occur during development. At the molecular level, the genomic DNA in each cell is composed of about 3.2 × 10^9^ base pairs. Given the estimated mutation rate of about 5 × 10^−11^ per nucleotide per replication [3], 0.16 mutations occur per genome during each cell division. At this rate, our body will have a total of more than 10^13^ (0.16 × 10^14^) mutations in a lifespan. In the context of cancer, 10^13^ will need some reductions, because most mutations will be neutral and multiple mutations should be accumulated to develop into malignancies. However, the magnitude of the initial number is certainly too high to become insignificant by such reductions. Under this mutation rate, nascent immortalized and/or transformed cells are postulated to arise incessantly. Therefore, the human body must have surveillance systems to monitor and eliminate such neoplastic cells before they are manifested into clinically detectable malignancies.

## 2. Classical Tumor Surveillance Mechanisms

Despite their significance, tumor surveillance mechanisms have garnered relatively less attention than other areas of oncology. In the cancer field, the term “surveillance” has usually been perceived as “immune surveillance”. In the immune surveillance hypothesis, the immune system can identify and eliminate cancer cells (reviewed in [4]). Although several studies (reviewed in [5]) have provided evidence for this hypothesis in the case of virally induced neoplasms, its universal role in all neoplasms is still questionable. In a broad sense, any mechanism that protects genomic/epigenomic integrity can also be regarded as tumor surveillance. For example, the proofreading of DNA polymerases and p53 play such roles during replication of the genome and DNA damage repair, respectively (reviewed in [6]). Although their malfunction ultimately leads to an elevated tumor rate, these mechanisms are technically “mutation surveillance” rather than “tumor surveillance.”

There are a number of cell death pathways that are a part of intrinsic biological programs or responses to external stimuli. Not surprisingly, these pathways are frequently deregulated in cancers. Most studies have focused on the inactivation of these pathways in cancer cells to explain their hyper-proliferative property (reviewed in [7]). However, it is conceivable that death pathways can play a tumor surveillance role, if they are activated selectively in neoplastic cells during tumorigenesis. This concept of “activation of cell death in cancer”, albeit seemingly paradoxical, is reasonable in the sense of tumor surveillance. As a novel example for this idea, we will discuss nc886, a non-coding RNA, in the remainder of this review.

## 3. Facts about nc886 and Protein Kinase R (PKR) in Cancer

nc886 is a non-coding RNA, 102 nucleotides in length, that exists abundantly in the human cellular cytoplasm [8]. nc886 is ubiquitously expressed in normal tissues or non-malignant cell lines, but is suppressed in many cancer cells of various tissue origins. nc886’s identity has been controversial, and it has other aliases including pre-miR-886 [9] and vtRNA2-1 [10,11]. However, nc886 is barely processed into mature microRNAs and is mostly dissociated from the vault complex [8]. Instead, nc886 has been shown to be a natural RNA ligand and repressor of PKR (Protein Kinase RNA-activated), as indicated by PKR activation and consequent impaired cell proliferation when nc886 is artificially suppressed [8,12,13].

**Figure 1:**
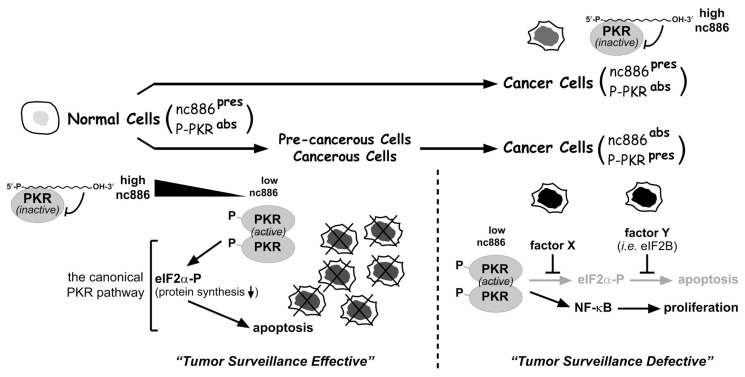
Illustration of the nc886-PKR tumor surveillance model. This cartoon has been modified from [12]. Active pathways are in black arrows; inactivated pathways are in grey arrows. Superscripted “pres” and “abs” indicate “present” and “absent”, respectively. See also the main text for the description of this illustration.

PKR is a sentinel kinase (reviewed in [14]). When triggered by dsRNA (double-stranded RNA) or other stimuli, PKR is auto-phosphorylated. Phospho-PKR (P-PKR in Figure 1) is the active kinase and phosphorylates eIF2α (eukaryotic initiation factor *2* α subunit). Phospho-eIF2α (eIF2α-P in Figure 1) inhibits global protein synthesis and ultimately induces cell death (“the canonical PKR pathway” in Figure 1). This PKR’s function is well established in the context of viral infection, which results in the production of dsRNA and consequent activation of PKR followed by cell death. Certainly the same scenario could be expected to occur during tumorigenesis when nc886 is suppressed.

## 4. A Tumor Surveillance Model Involving nc886 and PKR

The main frame of the model is that the suppression of nc886 in certain stages of tumorigenesis activates PKR and eliminates nascent transformed cells via the canonical PKR-eIF2α cell death pathway (see the “tumor surveillance effective” part in Figure 1). Obviously, cancer cells do develop and exist, indicating that they have escaped from the nc886-PKR sentinel (see the “tumor surveillance defective” part in Figure 1). Some cancer cells (nc886^present^, P-PKR^absent^ cancer cells in Figure 1) express nc886, indicating that nc886 has not been suppressed and thus PKR has not been released. Other cancer cells (nc886^absent^, P-PKR^present^ cancer cells in Figure 1) proliferate in spite of suppressed nc886 and activated PKR, by subverting the canonical PKR/eIF2α cell death pathway. In this case, PKR has responded to decreased nc886 but failed to eliminate the cell.

This model is supported by several pieces of experimental and circumstantial evidence. First, experiments in cholangiocarcinoma (CCA) have proven that the suppression of nc886 provokes the canonical PKR/eIF2α cell death pathway in non-malignant cholangiocyte cells, but not in CCA cells [12]. In some CCA cells, phosphorylated eIF2α is induced by PKR, but this does not inhibit protein synthesis. One molecular mechanism in these CCA cells is to elevate the expression of eIF2B, which neutralizes the inhibitory effect of phosphorylated eIF2α on protein synthesis. In other CCA cases, eIF2α fails to be phosphorylated by PKR. Intriguingly, the eIF2α branch in the PKR pathway appears to be subverted selectively, as another downstream branch NF-κB is activated by PKR and plays a pro-proliferative role in the same CCA cells [12].

Second, nc886 is suppressed and PKR is activated in a number of cancer cell lines and clinical specimens, for example, in CCA, breast cancer, head-neck cancer, acute myeloid leukemia, ovarian cancer, lung cancer, *etc.* ([8,12,15]; references in [12]; YSL, unpublished data). Given that PKR has been thought to be pro-apoptotic and tumor-suppressive, the elevated expression and/or activity of PKR in diverse cancer cells have been a mystery. Now this paradox can be understood more reasonably in the context of the tumor surveillance model.

Certainly, the model in Figure 1 is far from complete and the sketch must be supplemented with many more details. For instance, only little is known about the regulation of nc886 expression, albeit its suppression is the first critical event in the model. Although DNA hypermethylation at the nc886 locus explains why nc886 is suppressed in acute myeloid leukemia [15], the initial cue for the methylation remains to be identified. Besides epigenetic regulation, involvement of various transcription factors in nc886 expression is highly plausible, as their binding sites are found at the nc886 locus (SHJ and YSL, unpublished). Other questions to be answered include: How nc886 inhibits PKR, why PKR fails to phosphorylate eIF2α in some cancer cells, *etc*. In regards to the detailed molecular mechanisms through which tumor surveillance actually operates, we speculate that there are myriads of ways in individual cancer cells that differ in their tissue origins, genetic/molecular backgrounds, *etc*.

## 5. Concluding Remarks

In summary, we have introduced a novel type of tumor surveillance mechanism, which is primed by a non-coding RNA. We speculate that the nc886-PKR pathway is only one of many cell death pathways that play similar roles. Such pathways may have already been identified but disregarded because the activation of a cell death pathway in cancer is counter-intuitive. It will be worth revisiting those pathways with a view of the tumor surveillance model suggested here. Understanding an accurate role of a cell death pathway in cancer is also important in the clinic because it will provide better therapeutic strategies that selectively eliminate cancer cells.
